# Interhospital transfer of patients in extracorporeal membrane oxygenation

**DOI:** 10.1186/cc14356

**Published:** 2015-03-16

**Authors:** F Socci, S Di Valvasone, M Ciapetti, A Franci, M Bonizzoli, G Cianchi, S Batacchi, A Peris

**Affiliations:** 1 Careggi Hospital, Firenze, Italy

## Introduction

The transfer of patients in extracorporeal membrane oxygenation (ECMO) from a peripheral hospital to a tertiary center represents a high-risk situation of adverse events [1]. The aim of this retrospective study is to determine the feasibility and safety of interhospital transfer for critically ill patients with ECMO support.

## Methods

We collected data for the ECMO Regional Reference Centre Careggi Hospital activity from September 2009 to June 2014. In this study, 57 transfers were examined. The ECMO service is activated by a telephone call from a peripheral hospital. The team is represented by an intensivist, a heart surgeon, a cardiologist, a perfusionist and an intensive care nurse, all previously trained in the management of patients with ECMO. Medical personnel and the necessary equipment are transported by an ambulance and a van, specially designed and equipped for the transfer of patients with ECMO.

## Results

In this study, 57 patients transferred from the peripheral hospital to the ECMO center were examined; in all cases the ECMO system was implanted in the peripheral hospital (54 venovenous ECMO and three venoarterious ECMO). On average, trails were 271 km ± 304 (round trip) (minimum 14 km to maximum 939 km). The activation time from the call to the ambulance departure from our hospital was an average of 2 hours 27 minutes 13 seconds ± 1 hour 25 minutes 35 seconds. Transfer duration (from activation to return to the ECMO center) was an average of 8 hours 25 minutes 6 seconds ± 3 hours 27 minutes 58 seconds (minimum 3 hours to maximum 16 hours 55 minutes). The stop time (necessary for evaluation of the patient and for placement of the ECMO system) was an average of 3 hours 53 minutes 40 seconds ± 1 hour 6 minutes 35 seconds (minimum 2 hours 5 minutes to maximum 7 hours 30 minutes). Major complications related to malfunctions of the devices during transport were not recorded; in some cases it was necessary to manage minor complications (circuit cavitation, minor vascula accesses bleeding).

## Conclusion

Some studies have found several complications during transfer of patients in ECMO [2]. In our experience, there were no complications during the transfer of ECMO patients, even for longer trips. A wide and thorough clinical evaluation and multidisciplinary ECMO team allowed the optimization of clinical parameters before transport and a safely transfer. The start of ECMO treatment at peripheral hospitals and the transfer of patients in ECMO may be a viable option compared with conventional ventilation. Our data suggest that ECMO can be set up safely in peripheral hospitals by a multidisciplinary highly specialized ECMO team [3].
